# Violence, insecurity, and the risk of polio: A systematic analysis

**DOI:** 10.1371/journal.pone.0185577

**Published:** 2017-10-11

**Authors:** Kia Guarino, Arend Voorman, Maxime Gasteen, Donte Stewart, Jay Wenger

**Affiliations:** 1 Department of International Health, Johns Hopkins Bloomberg School of Public Health, Baltimore, Maryland, United States of America; 2 The Bill and Melinda Gates Foundation, Seattle, Washington, United States of America; University of Liverpool, UNITED KINGDOM

## Abstract

**Background:**

Since the introduction of polio vaccines in the 1950’s and 60’s, eradication of poliovirus from the world has been technically feasible. Progress towards this goal, however, has been uneven and influenced by social and political factors that challenge the implementation of robust immunization programs. While violence and insecurity are often cited as barriers to eradication, current global risk models are largely based on virologic and immunologic indicators measured at national levels. In this manuscript, we quantify the relevance of indicators of violence and insecurity on the risk of polio spread.

**Methods and findings:**

Using logistic regression models and public data sources, we evaluate the relationship between measures of violence and instability and the location of poliomyelitis cases between 2006 and 2015 at the country-level, both individually and after controlling for more proximal determinants of disease, such as nearby circulating poliovirus and vaccination rates. We found that increases in a country’s Fragile States Index (FSI) and Global Peace Index (GPI), aggregate indicators of violence and instability, were associated with the occurrence of poliovirus cases in the subsequent year (p< 0.01), even after controlling for established risk factors. These effects of violence and insecurity must be mediated through immunity and exposure to poliovirus, coarse measures of which are included in our model. This also implies that in our study, and in risk models in general, the interpretation depends on the quality and granularity of available data.

**Conclusion:**

National virologic and immunologic indicators understate the risk of poliovirus spread in areas with violence and insecurity, and the inclusion of such factors improves precision. In addition, the link between violence and incidence of disease highlights the broader challenge of implementing health interventions in conflict areas. We discuss practical implications of this work in understanding and measuring the risks to polio eradication and other global health initiatives, and the policy implications of the need to reach vulnerable populations in conflict zones.

## Introduction

Poliomyelitis (“Polio”) is an infectious viral disease which results in irreversible acute paralysis in up to 1% of infected people [1]. In 1988, the Global Polio Eradication Initiative (GPEI) developed a strategic plan to eradicate polio by 2000 [2]. A wide range of factors has delayed this timeline, but improvements in implementation tactics and vaccine technology (such as the use of monovalent and bivalent oral polio vaccine (OPV) formulations), have removed many of the technological obstacles.

**Fig 1:**
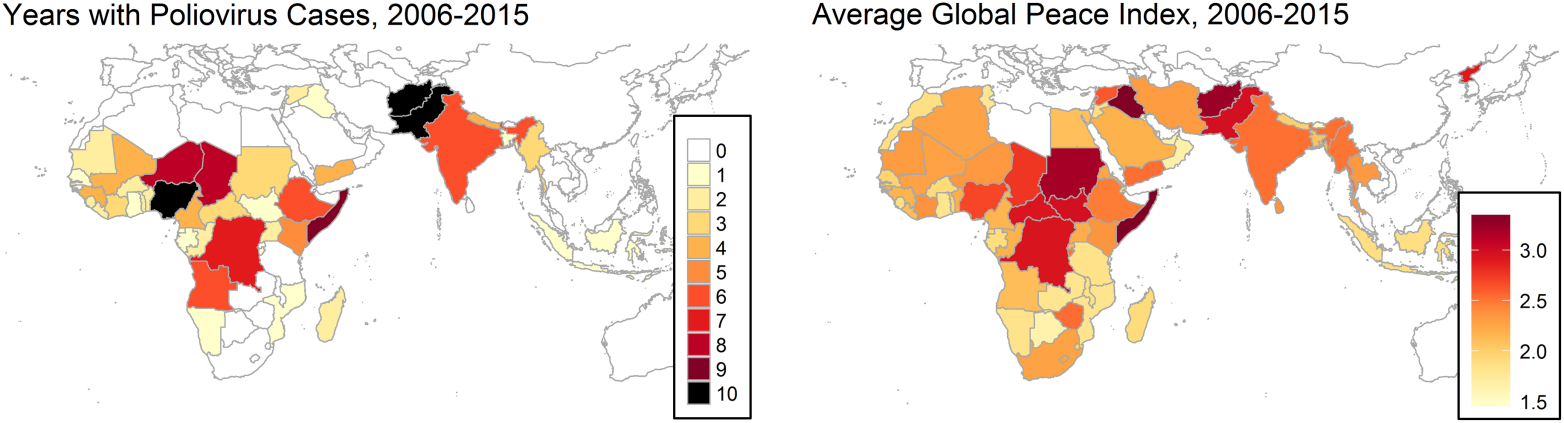
Geographic distribution of polio cases and Global Peace Index, 2006–2015.

Predictably, the last refuges of the virus are those areas with poorly functioning health systems and insecure environments, specifically Afghanistan, Pakistan, and Northern Nigeria. Reintroduction of virus with subsequent outbreaks have also occurred preferentially in insecure areas such as Syria and Somalia [3,4]. Insecurity affects the quality of service delivery and triggers mass population movement, making it challenging to access populations for vaccination campaigns. Countries with high scores on the Global Peace Index, a composite measure of peace using twenty-three qualitative and quantitative indicators, align roughly with countries that have had poliovirus cases over the last 10 years (Fig 1).

The risk of poliovirus spread has historically been understood from a classical infectious disease epidemiologic perspective, based on measures of immunity and proximity to poliovirus circulation [5,6]. While explicit threats to individual polio workers have been considered, broader violence and instability factors and their relationship to polio are not used in current risk models. This paper examines how physical and socio-political insecurity influence the risk of polio spread, and whether consideration of such factors augments a purely virologic/immunologic understanding of risks posed to eradication. Understanding these hurdles can help to better target human and financial resources to ensure timely disease eradication and to improve general health programs.

In some areas, the GPEI has faced specific threats and challenges to the polio program, such as vaccination bans and targeted violence against polio workers and the security forces that protect them [4,7–12]. A study by Kennedy, McKee, and King looks at the relationship between violence (measured as battlefield deaths), and polio cases [13]. Their findings suggest a link between post-2011 Islamist insurgencies and polio cases, although not between other conflicts and polio [13]. Another recent paper examined the relationship between Improvised Explosive Device (IED) detonations and polio, and found a strong association between sites of recent IED explosions and the location of polio cases in recent years in Afghanistan [14]. These authors conclude that knowledge of the geographic locations of violence can contribute to effective vaccination campaign planning as well as to the successful prediction of future polio incidence. However, while these studies associate different measures of violence with the existence of poliovirus, they do not do so in the context of all of the key known virological and immunologic risks for polio, such as vaccination status, making it impossible to determine whether the relationship identified is actually driven by other factors more relevant to the polio program. Our work extends these investigations by evaluating the relationship between incidence of polio and instability in the context of a broader epidemiological model of the risk of disease which includes infant mortality rate, routine immunization coverage, history of polio, and proximity to previous poliovirus circulation.

Our analysis primarily concerns the assessment of risk, rather than the physical process of disease spread and risk mitigation: the GPEI must systematically assess risks to the program and anticipate polio spread using available data, often coarsely measured at the national level. We recognize that conflict itself does not cause polio, but instead influences poliovirus spread by impacting other more proximal causes of the disease, such as vaccination rates. Thus, an association between conflict and polio in the context of a larger epidemiological model may improve our assessment of risk by highlighting deficiencies in available epidemiological data. How conflict impacts the process of disease spread and program implementation will vary widely with local context.

## Methods

A person is only able to acquire poliomyelitis if they are immunologically susceptible (i.e., do not have adequate antibodies to prevent infection and transmission, which is generally a function of vaccination status) and are exposed to poliovirus. As a result, the effects of violence and instability on the presence of polio cases must be mediated through one of these two factors. If susceptibility and poliovirus exposure are precisely and accurately known, then violence and instability are irrelevant to risk models of poliovirus spread. However, as will be demonstrated, susceptibility and poliovirus exposure are coarsely measured, especially in areas with violence and instability, and thus currently available data on these risk factors may not adequately predict future cases.

In light of these observations, this paper seeks to answer two specific questions:

1. Are violence and instability on their own predictive of whether a country will have future polio cases?
2. Do violence and instability indicate risk of poliovirus after adjusting for available measures of a country’s susceptibility and poliovirus exposure?

The first question addresses whether violence and political instability, or a correlate thereof, affect a country’s ability to immunize children or influence population movement leading to poliovirus transmission. In the second, we examine the more practical question of whether explicitly considering measures of violence and instability enhances our understanding of polio risk by indicating unmeasured susceptibility or exposure. In our statistical analyses, we are agnostic regarding the mechanism of these associations. However, we will discuss a few examples of how political instability may have impacted more proximal causes of the disease and led to observed differences in risk.

### Description of the data

Our analysis systematically assesses the relationship between the location of documented polio cases and intrinsic variables (previous poliovirus transmission, nearby transmission, and vaccination rates), public health and development indicators (infant mortality rate, immigration/emigration rates, access to improved water sources, and access to the internet), direct measures of violence (intentional homicide rate, and terrorism events), and broad indicators of political stability and violence (the Global Peace Index and the Fragile States Index). We limited our analysis to countries reporting to the African, Eastern Mediterranean, and South East Asian, regional offices of the World Health Organization (WHO) where polio was endemic during the period considered (2006–2015).

Data on recorded polio cases are available from the global polio surveillance network, which identifies acute flaccid paralysis (AFP) in children under the age of 15 [15]. Vaccine-induce childhood immunity estimates come from United Nations Children’s Emergency Fund (UNICEF)/WHO Estimates of National Immunization Coverage (WUENIC) [16]. Specifically, we used the coverage of the third dose of Diphtheria-Tetanus-Pertussis vaccine (DTP3 which is given at the same time as polio vaccine in the Expanded Programme on Immunization (EPI) schedule and is more reliably reported) as an indicator of childhood immunity to polio. In our primary analyses, we did not include a measure of Supplementary Immunization Activities (SIA), but we did include SIA impact in a sensitivity analysis which adjusted for reported SIA dose histories of non-polio AFP cases (though accuracy of such data for this purpose has been questioned [17]).

We used country-level population data from the United Nations 2015 Revision of World Population Prospects to account for differences in the total number of susceptible children in an area. We also used estimates of migrant stock, defined as the number of people born in a country different from where they live, to account for level of exposure of a population to those from other countries [18].

We used World Bank estimates of country-level Infant Mortality Rates (IMR) to adjust for overall health system quality[19]. Data from the World Bank on access to improved water sources was used as a general measure of level of development and as a factor contributing to poliovirus transmission [19].

We used the Fragile State Index (FSI), which assesses state vulnerability to conflict or collapse using 12 indicators, and the Global Peace Index (GPI) to capture additional political instability factors [20,21]. The FSI and GPI aggregate quantitative and qualitative indicators such as public perception of criminality, displaced people, and external conflicts from a variety of sources. The FSI is intended to measure a state’s vulnerability to conflict or collapse, while the GPI measures a state’s overall peacefulness. For both indicators, higher scores indicate more violence and instability. The GPI and the FSI are highly correlated (ρ = 0.8). We also used Intentional Homicides per 100,000 people and documented terrorist events to measure direct violence [19].

Certain country-level indicators, such as homicide rate, were not available for all countries in all years. Within a country, we accounted for missing values through linear interpolation, under the assumption that these indicators did not vary substantially from year-to-year. Even after interpolation, this missing data introduces measurement error and possible effect attenuation (i.e. bias towards the null hypothesis of no association). Table 1 gives a summary of these variables, for all countries and all years, as well as country-years with wild poliovirus (WPV) or vaccine derived poliovirus (VDPV) cases.

**Table 1:** Variable summary.

	All countries Median, IQR	Countries-years without WPV/VDPV cases	Countries-years with WPV/VDPV cases
**Countries (n)**	77	36	41
**Country-years (n)**	764	616	148
**WPV Cases**	0, (0–0)	-	8, (2–33.5)
**cVDPV Cases**	0, (0–0)	-	0, (0–2)
**DTP3 Coverage (%)**	87, (75–96)	91, (80–97)	72, (57–80.2)
**Infant mortality per 100,000**	45.6, (24.4–66.9)	40.7, (19.2–59.2)	70.8, (52.2–84.8)
**Fragile States Index**	87.3, (76.8–96.5)	83.9, (75.3–93.1)	99, (91.4–105)
**Global Peace Index**	2.13, (1.88–2.44)	2.05, (1.84–2.32)	2.51, (2.19–2.97)
**Terrorist events**	3, (1–22)	2, (1–13)	11, (2–174)
**Intentional homicides per 100,000**	5.58, (2.79–9.41)	4.77, (2.51–9.03)	6.69, (4.15–10.3)
**Migrant Stock (100,000s)**	2.53, (0.852–6.78)	2.42, (0.741–6.68)	3.54, (1.25–7.96)
**Access to improved water source (%)**	81.2, (62.4–92.7)	85.4, (71.8–95.7)	59.2, (50.3–74.9)

### Statistical analysis

We quantified the relationship between the various indicators and the presence of poliovirus cases using logistic regression models. In the first model, we used simple logistic regression model to test each variable for its association with future polio cases.

logit(Pr(caseinyearT))=α+β(variableinyearT−1),

where

logit(x)=log(x/(1−x)).

Our second model looks at whether each variable adds to a basic model of risk using intrinsic poliovirus epidemiology variables. The intrinsic epidemiologic variables we used in the basic model are (1) the presence of WPV/VDPV case in the previous year, (2) the presence of a WPV/VDPV case in a neighboring country in the previous year, (3) DTP3 coverage as a measure of childhood immunity and (4) population size. In Model 2, we examined every other variable individually to avoid co-linearity.

$$\begin{array}{ll} \text{logit}\left(\text{Pr}\left(\text{case}\;\text{in}\;\text{year}\;T\right)\right)= & \alpha +\beta_{1}\left(\text{case}\;\text{in}\;\text{year}\;T-1\right)+\beta_{2}\left(\text{Case}\;\text{in}\;\text{neighboring}\;\text{country}\;\text{in}\;\text{year}\;T-1\right)+\\ & \beta_{3}\left(\text{DTP3}\;\text{coverage}\;\text{in}\;\text{year}\;T-1\right)+\beta_{4}\left(\text{population}\;\text{in}\;\text{year}\;T-1\right)+\beta_{5}\left(\text{variable}\;\text{in}\;\text{year}\;T-1\right) \end{array}$$

In Model 3 we examined whether indicators of violence and instability add to a model based on an expanded set of covariates—those in Model 2 as well as infant mortality rate, migrant stock, and access to an improved water source. This model assesses whether measures of violence and instability indicate risk beyond what one would expect based on factors directly related to polio, as well as basic development and health-system indicators. We also fit an extension of model 3 with all covariates included, for the purposes of prediction.

Note that in Models 2 and 3 we adjust for serial correlation of the outcomes by including lagged terms. This is not done in Model 1, since the goal is to assess univariate associations. We also fit a model which corrects for this, and find that it does not materially alter the conclusions (see S1 File).

In all hypothesis tests, we used p < 0.05 to declare a result ‘statistically significant’, where we tested against the null-hypothesis of no effect (odds ratio = 1.0). Applied to the four indicators of violence and instability tested in three models, the expected number of false positives when all null-hypothesis are true is 0.6. All analyses were carried out in R [22]. The data and code used for the analysis are available in the S2 and S3 Files.

In addition to Models 1–3, we also fit several sensitivity analyses. We performed all analyses described above, where we omitted all cVDPV cases and only considered wild poliovirus cases. We also considered additional measured of vulnerability, such as dose histories of non-polio AFP cases. We also fit the model separately for the periods 2006–2011 and post-2011 by including an interaction term for each covariate and the indicator of whether the year analyzed was after 2011. The linear term corresponding to each variable *z* takes the form

β×z+β*×I(year>2011)×z,

and so the coefficient for *z* in the pre-2011 years is *β*, and for the post 2011 years is *β* + β*. A test for whether *β* or the linear combination *β* + β* were different from zero indicates whether there is evidence for an association in the pre and post 2011 period, respectively, while a test of whether β* is different from zero indicates whether there is evidence for there being a different effect in the two periods.

## Results

Table 2 displays results from the statistical analysis. All variables in Model 1, with the exception of intentional homicide rate and migrant stock, show strong association with poliovirus cases in the subsequent year. Notably, increases in both FSI and GPI were strongly related to polio cases in the subsequent year (p < 10^−16^). The association between GPI and polio can be seen to some extent in the map in Fig 1, reiterating the common observation that recent polio cases have occurred largely in countries with security concerns.

**Table 2:** Statistical analysis results.

	Model 1 Simple Logistic Regression		Model 2: Adjusting for intrinsic polio variables		Model 3: All variables	
	Odds ratio (95% CI)	p-value	Odds ratio (95% CI)	p-value	Odds ratio (95% CI)	p-value
**WPV/VDPV in previous year**	28.3, (17.9, 44.7)	2.90E-46	9.9, (5.71, 17.16)	3.20E-16	7.6, (4.3, 13.4)	3.00E-12
**Neighboring country with WPV/VDPV in previous year**	9.60, (5.83, 15.8)	5.60E-19	2.83, (1.49, 5.37)	0.0015	2.2, (1.2, 4.3)	0.017
**DTP3 Coverage (%)**	0.93, (0.92, 0.94)	7.70E-25	0.96, (0.94, 0.97)	2.20E-08	0.98, (0.96, 0.99)	0.0059
**Population (millions)**	1.002, (1.001, 1.003)	0.00022	1.000, (0.999, 1.002)	0.3	1.000, (0.999, 1.002)	0.53
**Infant Mortality per 100,000**	1.05, (1.04, 1.06)	3.70E-23	1.02, (1.01, 1.04)	0.00049	1.02, (1.004, 1.03)	0.013
**Fragile States Index**	1.09, (1.07, 1.11)	5.90E-19	1.04, (1.01, 1.06)	0.0024	1.03, (1.01, 1.06)	0.017
**Global Peace Index**	9.11, (5.7, 14.56)	2.40E-20	2.55, (1.34, 4.84)	0.0044	2.55, (1.31, 5.00)	0.0061
**Terrorist events (1000s)**	4.00, (2.02, 7.91)	6.70E-05	1.81, (0.86, 3.81)	0.12	2.48, (1.08, 5.66)	0.031
**Intentional homicides (per 100,000)**	1.01, (0.98, 1.04)	0.55	0.98, (0.93, 1.03)	0.39	0.96, (0.9, 1.02)	0.22
**Migrant Stock (100,000s)**	1.00, (0.99, 1.02)	0.64	1.00, (0.98, 1.03)	0.7	1.03, (1, 1.05)	0.029
**Access to improved water source (%)**	0.94, (0.93, 0.95)	2.50E-22	0.97, (0.95, 0.99)	0.0012	0.97, (0.95, 0.99)	0.012

Results from Models 1, 2 and 3 are given from left to right. For each model, the rows of the variables that are common to all regressions performed for that model are shaded grey.

In Model 2, we find that after adjusting for intrinsic polio variables, most variables remain associated with polio cases in the subsequent year, although measures of direct violence (number of terrorist events and intentional homicide rate) do not. For those variables that remain associated, their odds ratios (ORs) are somewhat attenuated relative to those in Model 1, suggesting that some of the predictive power is due to their correlation with traditional epidemiological risk factors. Notably, both FSI and GPI remain highly predictive of future polio cases (p < 10^−5^), with increases in FSI and GPI again being associated with increased risk.

Lastly, in Model 3 all variables except intentional homicides and population appear associated with WPV and VDPV in subsequent years. This confirms the association between political instability and violence with disease, and shows that instability is related to features of risk that are not fully captured by coarse measures of susceptibility and poliovirus exposure, population movement (migrant stock), or a measure of sanitation (improved water source).

We also fit a model with all variables included, in which no measure of violence or instability reached statistical significance. This is due to co-linearity between different measures violence and instability. For instance, FSI and GPI are highly correlated (r = 0.67). While it is clear from Model 3 that measures of violence and instability are associated with risk of polio beyond what can be explained by other variables considered, we cannot separate their individual contributions. Nonetheless, the model improved fit relative to one without violence and instability (likelihood ratio test p-value = 0.037).

We also examined sensitivity of our model to an analysis using only WPV cases. In this model, the effect of neighboring cases appeared much stronger, giving odds ratios of 16.8, 5.7, and 4.7 in Models 1, 2, and 3, respectively, roughly doubles the odds ratios in Table 2. This is likely due to the fact that a WPV case must arise from contact with an individual infected with WPV, whereas VDPV cases can emerge without precedent. Associations for all variables were not materially different.

At the suggestion of a reviewer, we also included a history of outbreaks within the past 4 years, and the percentage of zero-dose and under-immunized (< 3 doses of OPV) non-polio AFP cases as intrinsic polio variables. While the presence of an outbreak in the last 4 years was associated with disease in the next year in Models 2 and 3, NP-AFP dose histories were not associated with disease in the following year after adjusting for DTP3 coverage. This is in line with Voorman and Lyons (2016) who showed systematic bias in the ascertainment of NP-AFP dose histories, and with O’Reilly *et al* (2017) who also found a lack of association [17,23]. In both cases, results for all other variables were not materially different (see S1 File).

Kennedy, McKee, and King suggest that violence played an influential role in polio occurrence following the killing of Osama bin Laden in 2011 but not before that time. To examine this, we also tested whether the character of the associations with indicators of violence and instability differed between the period before and after 2011. We did this by including an interaction term with measures of violence and instability (FSI, GPI, terrorist events, and intentional homicide rate) and an indicator for whether the year in consideration was after 2011. In Model 1, FSI, GPI, and terrorist events remained associated in both periods. In Model 2, FSI was significantly associated with polio in both periods, while the association with GPI was not statistically significant for the post-2011 period (p = 0.058). In Model 3, all measures of violence and instability were associated with cases after 2011, while only FSI and GPI were associated in the pre-2011 period. While terrorist events were significantly associated only in the post-2011 period, the interaction term was not statistically significant. Thus, the association did not reliably differ between the two periods. Overall, these results suggest that in both periods considered, instability and violence are associated with polio when considered alone (as in Model 1), and usefully augment a model based on traditional epidemiological variables (as in Models 2 and 3), suggesting a robust relationship that persists across time.

**Fig 2:**
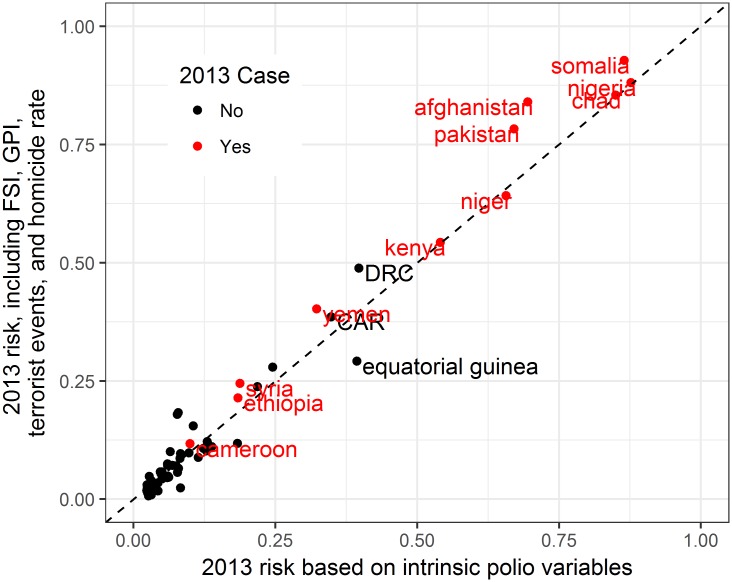
Estimated risk of polio in 2013 based on polio variables alone (X-Axis) compared to risk accounting for instability and violence (Y-Axis).

Fig 2 illustrates the predicted risk of a case in 2013, comparing a model with only intrinsic polio variables described above, and that same model including all variables related to violence and instability (FSI, GPI, terrorist events, and homicide rate). The plot shows how violence and instability might have altered our perception of risk of polio going into 2013, a year with numerous outbreaks. In the highest risk countries (Afghanistan, Pakistan, Somalia, Nigeria, Chad, and Niger), polio epidemiology alone was enough to predict occurrence of cases in 2013. However, inclusion of factors related to violence increase the accuracy of these predictions The plot also highlights uncertainty of predicting global polio epidemiology in general. For example, instability in Syria raised the risk for a case in 2013, but risk remained relatively low in both models given the lack of polio in the region.

## Discussion

Findings from our study suggest that political instability and violence are related to risk of wild and vaccine-derived polio cases in part through interaction with health systems and traditional epidemiological risk factors, and further, that violence and instability capture risk that is un-measured in a model based on traditional risk factors. While we do not explore the mechanism of this relationship, it is possible that violence and instability disproportionally affect certain sub-populations, leading to disruption of vaccination or population movement that are not represented by national epidemiological data. This is supported by the co-location of violent events and polio cases in sub-national areas, including northern Nigeria, southwestern Somalia, and along the Pakistan-Afghanistan border. Our work builds on previous studies by Norris and Kennedy by looking at the additional predictive value of insecurity measures, instead of focusing on the association between violence and polio [13,14]. However, the large number of variables comprising the GPI and FSI make it challenging to isolate the most significant contributing factors, requiring further research into the nuances of political instability.

This paper also supports existing research on how health systems functionality is impacted by violence and instability. The associations between polio and terrorist events, FSI, and GPI in Model 1 are present, but attenuated after adjusting for intrinsic polio variables in Model 2. That is, the associations between future incidence of polio and terrorist events, FSI, and GPI are explained in part by their association with vaccination coverage data and historical polio transmission. Whether their association could be fully explained by better or more granular coverage and epidemiological data, or whether violence and instability influence epidemiology through a separate mechanism is unclear. Additional insight may be gained by further research into how violence and instability affect both immunization rates more directly and disease outcomes in other programs such as regional Measles Elimination.

While our work highlights the importance of risk assessment in conflict affected countries, it should be complemented by research into how health programs can adapt and operate within these environments. Our data is limited to the past decade, but there is evidence that the modalities of conflicts have changed at least since the 1980s [24]. The proliferation of non-state actors and the power they exert both sub-nationally and across borders, challenge the traditional model of global health, which relies heavily on cooperation with sovereign states [25,26]. This operational model is hindered when non-state groups operate within sovereign territory and specifically target those delivering aid and services, as polio eradication groups have learned at great cost [27].

## Conclusions

We found that measures of political instability provide information about increased polio risk both on their own, and in addition to traditional technical and epidemiological risk factors. We conclude that instability and violence are related to the epidemiological characteristics that shape the geography of poliovirus transmission, and that explicitly incorporating measures of violence and instability into risk models improves understanding of poliovirus risk. However, identifying distinct causal relationships between violence, instability, health systems, and disease outbreaks remains challenging.

It is important to document the key lessons learned from global polio eradication, including those related to humanitarian access, negotiation, and innovative delivery approaches in complex settings such as Afghanistan, Somalia and Northern Syria. The broader global health and security communities need to engage more deeply with one another to address the challenge of delivering health care in conflict affected areas.

## Supporting information

S1 File Methods appendix. More detailed model description, and sensitivity analyses.
(DOCX)

S2 File Data. Final dataset used in the analysis.
(CSV)

S3 File Code. R code used to produce results of the analysis.
(R) Click here for additional data file.
