# Supplementary material for: Violence, insecurity, and the risk of polio: A systematic analysis
Source: PLoS One. 2017 Oct 11;12(10):e0185577. doi: 10.1371/journal.pone.0185577 (PMC5636089; doi:10.1371/journal.pone.0185577)
Supplement: S1 File — More detailed model description, and sensitivity analyses. (DOCX) [file pone.0185577.s001.docx]

**Note on serial dependence in the outcome variables**

Let $Y_{t}=\left\{ y_{1t}\ldots y_{Nt} \right\}$ be the vector of disease outcomes for countries $1\ldots N$ in year $t\in\{0,\ldots,T\}$, and let $x$ and $\theta$ be the covariates and associated parameters. Treating disease status in the first year $Y_{0}$ is fixed, iterative conditioning gives us

$$p\left( Y_{1},\ldots, Y_{T} | Y_{0};x, \theta\right)=\prod_{i=1\ldots T} p(Y_{t}|Y_{t-1};x, \theta),$$

where we model the conditional distributions $p\left( Y_{t} | Y_{t-1};x, \theta\right)$ using logistic regression, and fit the model with maximum likelihood. While the $Y_{t}$ are not strictly independent, the $Y_{t}|Y_{t-1}$ provide independent increments of information, and thus standard logistic regression procedures apply (see [1–3] for more general discussion). We also make the additional assumption that within a year, disease status between countries is independent (i.e. that $y_{jt}|Y_{t-1}$ is independent of $y_{kt}|Y_{t-1}$, for $j\neq k)$. This may be true to a first approximation, though there are circumstances in which it is not valid. For instance, a 2013 outbreak in the horn of Africa spread from Somalia to Kenya and Ethiopia within the same year.

Note that we do not adjust for serial correlation in the univariate analyses (Model 1). However, the purpose of that model is to examine the relationship between disease and insecurity in the absence of epidemiological indicators (i.e. without adjusting for disease status in the previous year). This model offers a useful comparison to show how covariates’ apparent effects change after adjustment. Thus inclusion of disease status in the previous year is not straightforward. However, serial correlation does not contribute significantly to the small p-values observed, and so we ignore it in the analysis for simplicity.

What follows is a technical discussion of how one could estimate univariate effects while accounting for serial correlation. Because this procedure is non-standard, difficult to interpret for a general scientific audience, and does not affect the conclusions, we omit it in the main manuscript.

Consider the variance estimate for the regression coefficient  $\hat{\beta}$in logistic regression

$Var(\hat{\beta)}\dot{=} \left( X^{T}diag\left( p\left( 1-p \right) \right)X \right)^{-1}$,

where $X$ is the matrix of regression coefficients and $p$ is the vector of fitted ‘success’ probabilities. Adjusting for serial dependence in Bernoulli random variables has the potential to inflate the variance of parameter estimates by reducing variability in the outcome. Loosely speaking, in the case of serial correlation after an observation we become more certain of the next observation in time. Inclusion of a lagged term thus drives the means $p$ closer to 0 or 1, making the elements of p(1-p) closer to 0, which tends to make $Var(\hat{\beta})$ larger. However, correlation between the lagged outcome and covariates will also change the estimates and interpretation of $\hat{\beta}$, and which may either increase or decrease associated p-values.

We want to assess the impact of a predictor of interest (GPI, FSI, etc) on polio incidence in a univariate sense, while accounting for the increased variance due to serial correlation. One way of doing this is by 1) projecting the lagged outcomes onto the orthogonal complement of the variable of interest, and then 2) performing the regression including these new lagged terms. In this way, we obtain the same fitted values $p$ as in the model that accounts for serial correlation (and the associated larger variance), but without competition between the variable of interest and the lagged variable. This is similar in spirit to ‘spatially restricted regression’ in [4], where the authors noted that spatially correlated errors interfere with covariates of interest in sometimes undesirable ways, and who suggested a similar strategy of adjustment. Formally, if $X$ is the matrix of covariates from Model 1 (i.e. the intercept term and the predictor of interest) and $z$ is the lagged outcome variable, we would replace $z$ with $z^{*}=\left( I-X\left( X^{T}X \right)^{-1}X^{T} \right)z$ , and likewise for the lagged indicator for neighboring cases. Note that this is an approximate procedure in logistic regression. Unlike in linear regression, $Var(\hat{\beta})$ need not be diagonal when covariates are orthogonal due to the influence of probability weights. This in turn implies that orthogonal covariates can still compete to a limited degree. Note also that the differences in fitted values influences regression coefficients in logistic regression, so that we do not expect exactly the same coefficients as in the univariate analysis, again unlike in linear regression with orthogonal covariates.

The table below compares the results of the procedure described above to that of simple logistic regression. While the confidence intervals are slightly wider using this procedure, and the p-values slightly larger, the results are not very different than the original analysis. R code for this analysis is included in the supplementary material.

|  | **Model 1 (as in manuscript)** | | **Model 1 (accounting for serial correlation)** | |
| --- | --- | --- | --- | --- |
|  | **Odds ratio (95% CI)** | **p-value** | **Odds ratio (95% CI)** | **p-value** |
| **WPV/VDPV in previous year** | 28.3, (17.9, 44.7) | 2.90E-46 | 33.97 (20.76, 55.58) | 9.40E-45 |
| **Neighboring country with WPV/VDPV in previous year** | 9.60, (5.83, 15.8) | 5.60E-19 | 9.47 (5.49, 16.34) | 6.90E-16 |
| **DTP3 Coverage (%)** | 0.93, (0.92, 0.94) | 7.70E-25 | 0.92 (0.91, 0.94) | 1.60E-24 |
| **Population (millions)** | 1.002, (1.001, 1.003) | 0.00022 | 1.002 (1.001, 1.004) | 9.80E-06 |
| **Infant Mortality per 100,000** | 1.05, (1.04, 1.06) | 3.70E-23 | 1.06 (1.04, 1.07) | 5.80E-20 |
| **Fragile States Index** | 1.09, (1.07, 1.11) | 5.90E-19 | 1.09 (1.07, 1.11) | 1.10E-14 |
| **Global Peace Index** | 9.11, (5.7, 14.56) | 2.40E-20 | 14.1 (7.79, 25.51) | 2.30E-18 |
| **Terrorist events (1000s)** | 4.00, (2.02, 7.91) | 6.70E-05 | 5.09 (2.35, 11.03) | 3.80E-05 |
| **Intentional homicides (per 100,000)** | 1.01, (0.98, 1.04) | 0.55 | 1.01 (0.97, 1.06) | 0.63 |
| **Migrant Stock (100,000s)** | 1.00, (0.99, 1.02) | 0.64 | 1 (0.99, 1.02) | 0.75 |
| **Access to improved water source (%)** | 0.94, (0.93, 0.95) | 2.50E-22 | 0.93 (0.91, 0.94) | 6.20E-19 |

**Impact of additional epidemiological and virologic indicators**

We chose a relatively simple range of epidemiologic and virologic indicators based on expert opinion, interpretability, and availability. The table below includes zero dose and under-immunized fractions of NP-AFP cases and the history of importation as ‘intrinsic polio variables’ in a sensitivity analysis, as suggested by a reviewer. The results of this analysis are nearly identical to that of our primary analysis, and do not change the interpretation. Relative to our original analysis, the association between FSI and GPI is slightly stronger in this analysis for both Models 2 and 3, while the association with terrorist events is slightly weaker in Model 3.

|  | **Model 1**  **Simple Logistic Regression** | | **Model 2:**  **Adjusting for intrinsic polio variables** | | **Model 3:**  **All variables** | |
| --- | --- | --- | --- | --- | --- | --- |
|  | **Odds ratio (95% CI)** | **p-value** | **Odds ratio (95% CI)** | **p-value** | **Odds ratio (95% CI)** | **p-value** |
| **WPV/VDPV in previous year** | 28.26, (17.86, 44.7) | 2.90E-46 | 10.61, (5.98, 18.81) | 6.40E-16 | 8.19, (4.53, 14.81) | 3.40E-12 |
| **Neighboring country with WPV/VDPV in previous year** | 9.6, (5.83, 15.8) | 5.60E-19 | 3.18, (1.66, 6.1) | 0.00051 | 2.46, (1.25, 4.82) | 0.0088 |
| **OB within last 4 yrs** | 0.93, (0.55, 1.55) | 0.77 | 0.39, (0.2, 0.76) | 0.0059 | 0.42, (0.22, 0.84) | 0.013 |
| **DTP3 Coverage (%)** | 0.93, (0.92, 0.94) | 7.70E-25 | 0.96, (0.94, 0.97) | 9.20E-07 | 0.98, (0.96, 0.99) | 0.012 |
| **Zero-dose (%)** | 1.03, (1.01, 1.05) | 0.0038 | 1, (0.96, 1.04) | 0.91 | 1.01, (0.96, 1.05) | 0.83 |
| **Under-imm. (%)** | 1.03, (1.01, 1.04) | 2.60E-05 | 1, (0.97, 1.03) | 0.8 | 0.99, (0.96, 1.03) | 0.76 |
| **Population (millions)** | 1, (1, 1) | 0.00022 | 1, (1, 1) | 0.5 | 1, (1, 1) | 0.7 |
| **Infant Mortality per 100,000** | 1.05, (1.04, 1.06) | 3.70E-23 | 1.02, (1.01, 1.04) | 0.0005 | 1.02, (1, 1.03) | 0.016 |
| **Fragile States Index** | 1.09, (1.07, 1.11) | 5.90E-19 | 1.05, (1.02, 1.07) | 0.00055 | 1.04, (1.01, 1.07) | 0.0056 |
| **Global Peace Index** | 9.11, (5.7, 14.56) | 2.40E-20 | 2.93, (1.49, 5.77) | 0.0019 | 2.89, (1.42, 5.88) | 0.0034 |
| **Terrorist events (1000s)** | 4, (2.02, 7.91) | 6.70E-05 | 1.74, (0.8, 3.76) | 0.16 | 2.3, (0.99, 5.38) | 0.054 |
| **Intentional homicides (per 100,000)** | 1.01, (0.98, 1.04) | 0.55 | 0.98, (0.93, 1.03) | 0.47 | 0.97, (0.91, 1.03) | 0.27 |
| **Migrant Stock (100,000s)** | 1, (0.99, 1.02) | 0.64 | 1, (0.98, 1.03) | 0.83 | 1.02, (1, 1.05) | 0.054 |
| **Access to improved water source (%)** | 0.94, (0.93, 0.95) | 2.50E-22 | 0.97, (0.95, 0.99) | 0.00095 | 0.97, (0.95, 0.99) | 0.013 |

The lack of association between NP-AFP dose histories in Models 2 and 3 (i.e. after adjustment) is in line with O’Reilly *et al* (2017), who also do not find an association. As with their paper, we also find that having an outbreak in the last 4 years is significantly associated with subsequent infection [5].

**References**

1. Bonney GE. Logistic Regression for Dependent Binary Observations. Biometrics. 1987;43: 951–973.

2. Cox DR, Snell EJ. The Analysis of Binary Data. 2nd ed. London, UK: Methuen; 1989.

3. Liang K, Zeger SL. A Class of Logistic Regression Models for Multivariate Binary Time Series. J Am Stat Assoc. 1989;84: 447–451.

4. Hodges JS, Reich BJ. Adding spatially-correlated errors can mess up the fixed effect you love. Am Stat. Taylor & Francis; 2010;64: 325–334.

5. O’Reilly KM, Lamoureux C, Molodecky NA, Lyons H, Grassly NC, Tallis G. An assessment of the geographical risks of wild and vaccine-derived poliomyelitis outbreaks in Africa and Asia. BMC Infect Dis. 2017;17. doi:10.1186/s12879-017-2443-4
